# Isolation of Novel Probiotic *Lactobacillus* and *Enterococcus* Strains From Human Salivary and Fecal Sources

**DOI:** 10.3389/fmicb.2020.597946

**Published:** 2020-12-04

**Authors:** Homa Bazireh, Parvin Shariati, Sadegh Azimzadeh Jamalkandi, Ali Ahmadi, Mohammad Ali Boroumand

**Affiliations:** ^1^Department of Bioprocess Engineering, Institute of Industrial and Environmental Biotechnology, National Institute of Genetic Engineering and Biotechnology, Tehran, Iran; ^2^Chemical Injuries Research Center, Systems Biology and Poisonings Institute, Baqiyatallah University of Medical Sciences, Tehran, Iran; ^3^Molecular Biology Research Center, Systems Biology and Poisonings Institute, Baqiyatallah University of Medical Sciences, Tehran, Iran; ^4^Department of Pathology and Laboratory Medicine, Tehran Heart Center, Tehran University of Medical Sciences, Tehran, Iran

**Keywords:** probiotics, saliva, feces, *Lactobacillus*, *Enterococcus*, microbiome, lactic acid bacteria

## Abstract

Probiotics are non-pathogenic microorganisms that can interact with the gastrointestinal microbiota. They have numerous beneficial health effects that include enhancement of the host immune response, antiallergic, antimicrobial, anti-cancer, and anti-inflammatory properties. Probiotics are capable of restoring the impaired microbiome of a dysbiotic gut. They can be isolated from different environments. However, it is frequently suggested that probiotics for human use should come from human sources. The objective of this study was to isolate and characterize novel probiotic strains from the saliva and feces of healthy human individuals. To meet the criteria for probiotic attributes, the isolates were subjected to numerous standard morphological and biochemical tests. These tests included Gram staining, catalase tests, antibiotic susceptibility testing, hemolytic and antagonistic evaluation, tolerance tests involving temperature, NaCl levels, pH and bile salts, adherence ability assays, and genotypic characterization involving 16S rRNA gene sequencing. From 26 saliva and 11 stool samples, 185 microbial strains were isolated. Based on morphological and biochemical characteristics, 14 potential probiotic candidates were selected and identified genotypically. The new strains belonged to *Lactobacillus fermentum, Enterococcus faecium*, and *Enterococcus hire*. The selected strains were non-hemolytic, showed high tolerance to low pH and bile salts, and strong adherence abilities. Furthermore, the strains displayed a wide range of antimicrobial activities, particularly against antibiotic-resistant pathogens such as methicillin resistant *Staphylococcus aureus* (MRSA). Moreover, five of the selected isolates demonstrated antiproliferative features against human colon cancer cell line (Caco-2). The results of this investigation confirm the diversity of microbial populations in the human gut and saliva, and since these strains are of human origin, they will highly likely display maximal activities in food and drugs set for human use. Hence, the new strains of this study require additional *in vivo* experiments to assess their health-promoting effects.

## Introduction

Probiotics are live microorganisms that can confer health benefits to the host when consumed in adequate amounts FAO/WHO (2002). In fact, probiotics have recently been developed that can balance and restore the human gut microbiome inflicted with dysbiosis (Kumar et al., 2020). Many probiotics are lactic acid-producing bacteria (LAB) that are Gram-positive and catalase-negative. The two most common genera of probiotics are *Lactobacillus* and *Bifidobacterium*, which have been shown to have beneficial roles in human health.

The use of probiotics not just as supplements but as actual treatment strategies for various diseases has now become more prevalent and the current focus of attention in the scientific and medical communities. The multidimensional effects of probiotics are currently being evaluated in many fields of medicine that include infectious diseases (Anwar et al., 2020; d’Ettorre et al., 2020; Silva et al., 2020; Tan et al., 2020), the immune system (Gill et al., 2000; Dargahi et al., 2020), chronic diseases such as cancer (Haghshenas et al., 2014; Nami et al., 2015), cardiovascular (Ettinger et al., 2014; Gómez-Guzmán et al., 2015; Daliri et al., 2017), neurodegenerative (Westfall et al., 2017), inflammatory diseases (Plaza-Díaz et al., 2017), and diabetes (Kocsis et al., 2020).

Accordingly, this has culminated in the search for new bacterial strains with numerous inherent attributes that can be of potential use in the treatment of many ailments and disorders. For example, the localized use of the probiotic *Lactobacillus plantarum* ATCC 10241 strain in a burn model was found to interfere with *Pseudomonas aeruginosa*, stimulating phagocytosis of this pathogen by tissue phagocytes, decreasing apoptosis, and thereby improving tissue repair (Valdéz et al., 2005). In recent years with the emergence of antibiotic resistance, a lot of emphasis has been placed on investigating probiotics and their products as alternatives to antibiotics. The antagonistic activity of probiotics against pathogens is brought about by a series of mechanisms that include, competitive exclusion of pathogens, boosting the function of the intestinal barrier, and producing effective antimicrobial compounds such as peptides (Fijan, 2016; Besser et al., 2019). Hence, numerous *Lactobacillus* strains have been found to inhibit the growth of many different types of multi-drug resistant bacterial pathogens, such as MRSA (methicillin resistant *Staphylococcus aureus*), *Streptococcus mutans*, *Escherichia coli*, *P. aeruginosa*, *Klebsiella pneumoniae*, *Shigella* spp. and *Clostridium difficile* (McFarland, 2015; Kumar et al., 2016; Kang et al., 2017; Chen et al., 2019; Nami et al., 2019b).

In another study, the cholesterol removal capacity of *L. plantarum* YS5 *in vitro* was shown to reduce cholesterol levels by 84%. Moreover, probiotic supplementation was found to decrease serum total cholesterol, low density lipoprotein cholesterol, and triglyceride levels in male Wistar rats (Nami et al., 2019a). In other research involving colon cancer, the administration of *Lactobacillus acidophilus* ATCC 314 and *Lactobacillus fermentum* NCIMB 5221 in the murine colon cancer model was found to reduce or stop the growth of tumors, by stimulating an antitumor immune response (Kahouli et al., 2017). Many studies have shown that certain specific probiotics exhibit anticarcinogenic activities and contribute to the prevention of colon cancer through host-dependent mechanisms. One such mechanism involves the production of metabolites such as SCFAs, acetate, propionate and butyrate by a number of probiotic *Lactobacillus*, *Bifidobacterium*, and *Streptococcus* strains, showing positive effects on immune and epithelial cells (Ganapathy et al., 2013; Singh et al., 2014; Gao et al., 2017; Drago, 2019).

Probiotics can be found in many environments such as dairy products, fermented, food and humans. However, the use of probiotics of human origin for use in humans is frequently proposed (Sanders, 2008; Kumar et al., 2020).

The aim of this study is to identify novel indigenous bacterial strains from healthy human individuals that can be used as potential probiotics for the treatment and prevention of various human ailments. In order to be recognized as potential probiotics, bacterial strains that are isolated from various sources must meet specific criteria. For this purpose, a series of standard tests are usually carried out to identify and characterize potential probiotic strains. These tests include evaluating the ability to survive under harsh conditions, e.g., low pH, the presence of antibacterial activity, ability to adhere to epithelial cells, demonstrating non-hemolytic activity, and lacking antibiotic resistance genes.

## Materials and Methods

### Materials, Reagents and Strains

Standard strains were purchased from the Iranian Research Organization for Science and Technology (IROST), and culture media were obtained from Ibresco, Zist Kavosh Iranian Co, Iran. All antibiogram disks were provided by Padtan Teb Co. (Iran) including gentamycin, cefixime, penicillin, chloramphenicol, streptomycin, erythromycin, ampicillin, trimethoprim, kanamycin, vancomycin, rifampin, azithromycin, and clindamycin. Also, molecular detection was carried out using the PCR master mix kit (Ampliqon, Denmark), and primers (synthesized by Taq Copenhagen Co, Denmark). For cell culture experiments, reagents were obtained from DNAbiotech Co. (Iran).

### Sampling

Twenty six saliva and 11 stool samples were collected from healthy human individuals. People were informed regarding the study, and written consent forms were provided. This study was approved by the ethics committee at the National Institute of Genetic engineering and Biotechnology (NIGEB, Tehran, Iran) and registered as IR.NIGEB.EC.1398.12.3.B. Samples were transported to the laboratory on ice and were immediately diluted with peptone water, spread onto de Man-Rogosa-Sharpe (MRS) agar medium and Brain Heart Infusion (BHI) agar, then incubated for 48–72 h at 37°C under aerobic and microaerophilic (by using an anaerobic jar) conditions.

### Biochemical and Morphological Characterization

Morphological characterization was carried out using the Gram staining technique, and biochemical characterization was performed using the catalase test and analysis of carbohydrate fermentation profiles. Physiological tests included the ability to grow in the presence of NaCl [3% and 4.5% (w/v)], and also at temperatures of 15°C and 45°C. All catalase-negative and Gram-positive bacilli or cocci, the morphology of which was similar to LAB bacteria were classified as potential probiotic strains.

### Hemolytic Activity

Fresh bacterial cultures were streaked onto blood agar media [containing 5–10% sheep blood (Zist Royesh Co, Iran)] and incubated for 24 h at 37°C. The isolates were then examined for the presence of clear zones surrounding the colonies. Clear zones are considered as beta hemolysis, greenish zones as alpha hemolysis and the absence of zones indicating no hemolysis is known as gamma hemolysis. Colonies showing beta or alpha hemolysis were excluded, and only those with gamma hemolysis were selected (Halder et al., 2017).

### Survival Under Low pH

In order to determine the acid tolerance of the bacterial isolates, a procedure was carried out in accordance with standard protocols, but with some minor modifications (Vernazza et al., 2006; Haghshenas et al., 2016). Briefly, fresh overnight bacterial cells were harvested by centrifugation and inoculated at 1% (v/v) into MRS broth (pH 3). The cultures were incubated for 3 h at 37°C. Thereafter, culture samples were removed at 0 and 3 h, and spread onto MRS agar plates, which were then incubated at 37°C. Survival rate was measured at 0 and 3 h after incubation using the colony count procedure.

### Bile Salt Tolerance

This test was conducted according to the method by Nami et al. (2019b), but with some minor changes. In brief, overnight bacterial cultures were inoculated at 1% (v/v) into both MRS broth media (control) and MRS broth containing 0.3% (w/v) oxgall (Ibresco Co, Iran). They were both incubated for 4 h at 37°C, and the optical density (OD) of the cultures was then measured at 600 nm. Subsequently, the percentage of growth inhibition was determined with the following formula:

(1) Inhibition%=(Growth in control−Growth in oxgall/Growth in control)×100

### Antagonistic Activity Against Pathogens

To detect the LAB inhibitory properties against chosen pathogens, the well diffusion assay method was used (Alkalbani et al., 2019; Chen et al., 2019). Briefly, bacterial isolates cultured at 37°C for 24–48 h were centrifuged for 10 min at 10,000 rpm, and the resulting supernatants were then separated and used against ten pathogenic bacterial and fungal strains including, *S. aureus* ATCC 25923, *Salmonella enterica* ATCC 14028, *P. aeruginosa* ATCC 27853, Methicillin-resistant *Staphylococcus aureus* (MRSA) ATCC 33591, *Escherichia coli* (*E. coli*) ATCC 25922, *S. mutans* ATCC 35668, *Listeria monocytogenes* ATCC 13932, *Bacillus cereus* ATCC 11778, *Enterococcus faecalis* ATCC 29212, and *Candida albicans* ATCC 10231. After 24 h of incubation, the inhibition zones around the wells were measured. Each test was conducted in triplicate.

### Antibiotic Susceptibility Test

The antibiotic susceptibility test was conducted using the disk diffusion assay method. Fresh overnight cultures of bacterial isolates were spread onto MRS or BHI agar plates, and 13 antibiogram disks were then carefully placed on the agar plates, which were subsequently incubated at 37°C for 24 h. The antibiotic disks consisted of gentamycin (10 μg), cefixime (5 μg), penicillin (10 μg), chloramphenicol (30 μg), streptomycin (10 μg), erythromycin (15 μg), ampicillin (10 μg), trimethoprim (5 μg), kanamycin (30 μg), vancomycin (30 μg), rifampin (5 μg), azithromycin (15 μg), and clindamycin (2 μg). Finally, results were reported according to the Clinical and Laboratory Standards Institute (CLSI) guidelines (Kook et al., 2019).

### Adhesion Ability

The human colon carcinoma cell line (Caco-2; kindly provided by NIGEB) was grown in high glucose Dulbecco’s Modified Eagle’s Medium (DMEM; DNAbiotech Co, Iran) supplemented with 10% FBS and 1% Penicillin-Streptomycin, at 37°C with a 5% CO_2_ atmosphere. The medium was changed every other day until the cell confluency of 70–80% was reached. The cells were then trypsinized, counted (4 × 10^5^ cells/mL) and transferred to a 24-well dish. The absorbance of the freshly prepared bacterial cultures was adjusted to 0.5 McFarland using DMEM. Then, 100 microliters of bacterial suspension were added to each well followed by incubation at 37°C for 2 h. Afterward, cells were washed twice with phosphate buffer saline (PBS), fixed with methanol and stained with crystal violet for 5 min. Adherent cell numbers were counted as outlined previously by (Fernández et al., 2003).

### Molecular Identification

The bacterial isolates that fulfilled the selection criteria for probiotics were finally chosen as probiotic candidates to be identified genotypically using the 16S rRNA gene amplification method.

The PCR reaction mixture with a total volume of 25 microliters, consisted of 10 pmol primers, and the PCR master mix reaction mixture. The following universal primers; 27F (5′ AGA GTT TGA TCC TGG CTC AG 3′) and 1492R (5′ GGT TAC CTT GTT ACG ACT T 3′) were used in the reaction.

PCR program in the thermal cycler (peQlab, United States) was comprised of initial denaturation at 95°C for 10 min followed by 35 cycles containing the second denaturation at 95°C for 1 min, annealing at 60°C for 1 min and extension at 72°C for 1 min and 30 s, followed by a final extension step at 72°C for 10 min.

PCR products were detected and visualized by agarose gel electrophoresis (1% w/v) and subsequently sequenced. The resulting Sanger sequencing data were employed by the basic local alignment search tool (BLAST) to obtain sequence similarities. Thereafter, the sequences were registered in the NCBI^1^ database and assigned with accession numbers.

### Biofilm Production

The potential ability of probiotic strains to form biofilm was investigated, as previously reported by Pérez Ibarreche et al. (2014). After 24 h of incubation at 37°C, the OD of the isolates was measured at 570 nm using an ELISA microplate reader (Biotech, United States). Comparison of the strains with the negative control (MRS and PBS), revealed the strains competency in biofilm formation. Each test was conducted in three experiments and the final cut- off was considered as non-biofilm formation (OD ≤ ODc [ODc: the OD of the control]), week biofilm formation (ODc < OD ≤ 2 × ODc), modest biofilm formation (2 × ODc < OD ≤ 4 × ODc), and strong Biofilm formation (4 × ODc < OD; ODc is the optical density of the control; Borges et al., 2012).

### Auto-Aggregation

The auto-aggregation test evaluates the ability of isolates to adhere to the intestine, exerting antipathogenic effects (Krausova et al., 2019). This test was performed using the method by Xu et al. (2009). Fresh cultures of bacterial isolates (grown for 16–18 h) were washed twice with PBS, and the optical densities of the resulting bacterial suspensions were adjusted to 0.5 McFarland at 600 nm. They were subsequently incubated for 2 h at 37°C, thereafter the upper phase was removed, and its OD was measured. Finally, the auto-aggregation percentage was determined by using the following formula (A0 = Initial OD, At = OD after 2 h).

Auto-aggregation(%)=(ODA0-ODAt/A0)× 100

### Hydrophobicity

To further assess the adhesion abilities of the probiotic isolates, the hydrophobicity of the isolates was measured using the microbial adhesion to hydrocarbons (MATH) method, as described by Vinderola et al. (2004). In short, an overnight culture of the isolates was washed twice using PBS, and their optical densities were then adjusted to 0.5–0.6 at 600 nm (A0). One milliliter of xylene was added to each suspension and vortexed vigorously for 1 min. Then the mixture was incubated at 37°C for 1 h. After incubation and phase separation, the aqueous phase was carefully removed to measure its absorbance (At). Hydrophobicity percentage was calculated with the formula presented below.

Hydrophobicity(%)=(1-At/A0)× 100

### MTT Assay

The 3-(4,5-Dimethylthiazol-2-yl)-2,5-diphenyltetrazolium bromide (MTT) assay was conducted to show the cytotoxic effects of bacterial culture-free supernatants on CaCo-2 cell lines as pointed out by (Chen et al., 2017). In short, 10^4^ cells were seeded in 96 microtiter plates and were allowed to attach to the bottom of the plate. Then, different concentrations (25 and 100 microliters) of the fresh bacterial culture-free supernatants were added to each well. After 24, 48, and 72 h of incubation at 37°C with a 5% CO_2_ atmosphere, MTT solution was added to each well and the resulting mixtures were incubated for another 3–4 h. The solution in every single well was then collected following the addition of 100 microliters of dimethylsulfoxide (DMSO) to each well. Finally, the OD was measured using an ELISA microplate reader at 570 nm. Cell viability was determined according to the following formula:

Cellviability(%)=(ODtreat/ODcontrol)× 100

### Statistical Analysis

Statistical analyses were carried out using one-way analysis of variance (ANOVA) and SPSS software version 25. Each test was performed in triplicate.

## Results

### Biochemical and Morphological Test Results

As shown in Table 1 all the strains were Gram-positive and catalase-negative, and were able to grow in the presence of 3% (w/v), 4.5% NaCl (w/v) and at the high temperature of 45°C, while nine strains were not able to grow at 15°C. Sugar fermentation patterns confirmed that the rod-shaped isolates were likely to be *Lactobacillus* strains whereas the cocci belonged to *Enterococcus* genus.

**TABLE 1:** Results of the morphological and biochemical tests carried out for selected isolates.

Strains	SA 151	SA 135	SA 171	SA 139	SA 109	SA 110	SA 12	SA 16	ST 80	ST 13	ST 67	ST 126	ST 172	ST 179
Cell morphology	rod	rod	rod	rod	rod	rod	rod	rod	cocci	cocci	cocci	cocci	cocci	cocci
Gram	+	+	+	+	+	+	+	+	+	+	+	+	+	+
Catalase	−	−	−	−	−	−	−	−	−	−	−	−	−	−
Growth in presence of NaCl 3%	+	+	+	+	+	+	+	+	+	+	+	+	+	+
Growth in presence of NaCl 4.5%	+	+	+	+	+	+	+	+	+	+	+	+	+	+
Growth at 15°C	−	−	−	−	−	−	−	−	−	+	+	+	+	+
Growth at 45°C	+	+	+	+	+	+	+	+	+	+	+	+	+	+
Carbohydrate fermentation														
Glucose	+	+	+	+	+	+	+	+	+	+	+	+	+	+
Galactose	+	+	+	+	+	+	+	+	+	+	+	+	+	+
Maltose	+	+	+	+	+	+	+	+	+	+	+	+	+	+
Mannose	−	−	−	−	−	−	−	−	−	+	+	+	+	+
Manitol	+	+	+	+	+	+	+	+	+	+	+	+	+	−
Cellobiose	+	+	+	+	+	+	+	+	+	+	+	+	+	+
Rhamnose	−	−	−	−	−	−	−	−	−	−	−	−	−	−
L−Arabinose	+	+	+	+	+	+	+	+	+	+	+	+	+	−
Fructose	+	+	+	+	+	+	+	+	+	+	+	+	+	+
L−xylose	+	+	+	+	+	+	+	+	+	−	−	−	−	−
Sorbitol	+	+	+	+	+	+	+	+	+	−	−	−	−	+
Sucrose	+	+	+	+	+	+	+	+	+	+	+	+	+	+
Lactose	+	+	+	+	+	+	+	+	+	+	+	+	+	+
Inositol	+	+	+	+	+	+	+	+	+	+	+	+	+	+

### Hemolytic Activity Results

In terms of hemolytic activity, three strains exhibiting hemolytic activity (beta or alpha hemolysis) were excluded, and the rest, which showed non-hemolytic activity, were used for further experiments.

### Survival Under Low pH Conditions Results

Among the 185 isolates screened for low pH tolerance, 43 exhibited tolerance to pH 3. The colonies of the potential acid-tolerant isolates were then counted at 0 and 3 h after incubation in MRS agar at pH 3. Strains SA 135, ST 80, and SA 151 demonstrated relatively the highest rate of survival after 3 h of incubation. The results are shown in Table 2.

**TABLE 2:** Acid tolerance and survival rate of selected isolates under acidic conditions.

Strains	0 h CFU/ml	3 h CFU/ml	Survival rate (%)
SA 151	6.17 ± 0.1	6.01 ± 0.03	97.4%
SA 135	7.38 ± 0.01	7.33 ± 0.03	99.32%
ST 80	6.51 ± 0.01	6.46 ± 0.01	99.23%
SA 139	6.47 ± 0.1	6.17 ± 0.1	95.36%
SA 171	6.47 ± 0.1	5.60 ± 0.1	86.55%
SA 12	7.8 ± 0.06	7.3 ± 0.3	93.58%
SA 109	6.54 ± 0.06	5.17 ± 0.1	79.05%
SA 110	7.09 ± 0.08	5.87 ± 0.02	82.79%
SA 16	6.47 ± 0.03	5.54 ± 0.06	85.62%
ST 13	7.17 ± 0.1	6.17 ± 0.1	86.05%
ST 67	6.49 ± 0.01	6.30 ± 0.2	97.07%
ST 126	6.47 ± 0.1	5.30 ± 0.2	81.91%
ST 172	7.14 ± 0.03	5.65 ± 0.04	79.13%
ST 179	6.90 ± 0.05	5.47±+0.1	79.27%

*Values are mean ± standard deviation of triplicates.*

### Bile Salt Tolerance Results

**FIGURE 1:**
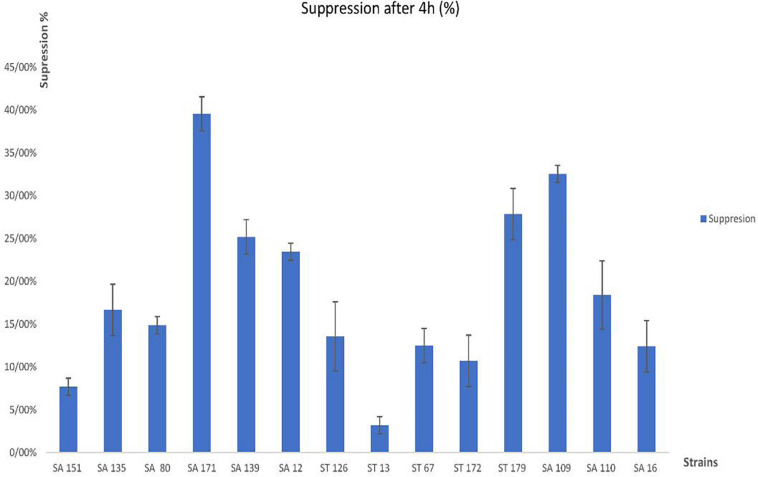
Percentage growth inhibition (suppression) of the 14 selected isolates by bile salts.

Following the assessment of bile salt tolerance for 4 h, isolates ST 13, SA 151, and ST 172 were shown to be the most resistant, with growth inhibition capabilities ranging from 3.21 ± 0.01% to 10.71 ± 0.03%, however, isolates SA 171, SA 109, and SA 179 exhibited the least resistance, ranging from 39.56 ± 0.02% to 27.85 ± 0.03%. In general, nearly all the strains showed above the 50% tolerance ability (Figure 1).

### Antagonicity Test Results

The isolated strains were assessed for antimicrobial activity against ten types of pathogens. The results are indicated in Table 3. Certain isolates had an inhibitory impact on the selected pathogens. The four isolates, SA 151, SA 135, ST 80, and SA 139 were able to moderately constrain a wide array of pathogens. Seven isolates including SA 171, SA 12, SA 109, SA 110, SA 16, ST 13, and ST 126 were able to inhibit at least four types of pathogens. Six isolates (SA 151, SA 135, ST 80, SA 109, SA 110, and SA 16) could inhibit the MRSA even though none inhibited the *S. enterica* strain.

**TABLE 3:** Antimicrobial activities against ten pathogens.

Strains	*S. aureus (mm)*	*L. monocytogenes (mm)*	*B. cereus (mm)*	*S. mutans (mm)*	*E. coli (mm)*	*P. aeruginosa (mm)*	*C. albicans (mm)*	*E. faecalis (mm)*	MRSA *(mm)*	*S. enterica (mm)*
SA 151	15	13	15	10.5	−	15.5	15.5	11	20	−
SA 135	15	15	16	15	−	17	20	−	11	−
ST 80	12	16	18	11	−	15	22	−	17	−
SA 139	–	10	13	15	10	13	16	10	−	−
SA 171	18	−	15	15	−	20	19	−	−	−
SA 12	−	−	14	12	−	10	12	12	−	−
SA 109	−	−	10	12	−	15	21	−	30	−
SA 110	−	−	10	10	−	12	20	−	25	−
SA 16	−	−	12	12	−	14	15	−	21	−
ST 13	−	14	10	−	14	12	−	10	−	−
ST 67	−	13	9	−	−	−	−	10	−	−
ST 126	−	13	13	−	15	17	−	15	−	−
ST 172	−	−	−	−	−	10	−	10	−	−
ST 179	−	10	−	−	−	−	−	−	−	−

*The zone diameter values above are the average of two experiments and presented in millimeter (mm).*

### Evaluation of Antibiotic Susceptibility

As shown in Table 4, antibiotic susceptibility tests using the disk diffusion method indicated that all the strains were resistant to kanamycin and streptomycin, except for SA 109, which was sensitive to streptomycin; nevertheless, each of the isolates was sensitive to chloramphenicol and ampicillin. No antibiotic resistance patterns were reported for erythromycin and clindamycin except intermediate susceptibility in (SA 139, SA 12, and ST 126) and (ST 80 and ST 179), respectively. Strains were resistant to gentamycin (*n* = 5), cefixime (*n* = 6), penicillin (*n* = 3), trimethoprim (*n* = 9), vancomycin (*n* = 8), rifampin (*n* = 6), and azithromycin (*n* = 1). Resistance rate, calculated via the number of antibiotic resistance of each strain to the whole number of tested antibiotics, varied from 23.07% (represented by SA 151, ST 110, and ST 16) to 46.15% (demonstrated by SA 139, ST 13).

**TABLE 4:** Antibiotic susceptibility test results.

Strains	Gentamycin	Cefixime	Penicillin	Chloramphenicol	Streptomycin	Erythromycin	Ampicillin	Trimethoprim	Kanamycin	Vancomycin	Rifampin	Azithromycin	Clindamycin
SA 151	S	S	S	S	R	S	S	S	R	R	S	S	S
SA 135	R	R	S	S	R	S	S	R	R	R	S	S	S
ST 80	R	R	S	S	R	S	S	R	R	R	S	S	I
SA 171	S	S	R	S	R	S	S	S	R	R	S	S	S
SA 139	R	R	S	S	R	I	S	R	R	S	R	I	S
SA 109	S	R	S	S	S	S	S	R	R	R	S	S	S
SA 110	S	S	S	S	R	S	S	S	R	R	S	S	S
SA 12	R	I	S	S	R	I	S	R	R	S	R	I	S
SA 16	S	S	S	S	R	S	S	S	R	R	S	S	S
ST 13	S	R	R	S	R	S	S	R	R	S	R	S	S
ST 67	S	I	R	S	R	S	S	R	R	S	R	S	S
ST 126	S	S	R	S	R	I	S	R	R	S	R	S	S
ST 172	S	I	S	S	R	S	S	R	R	S	R	R	S
ST 179	R	R	S	S	R	S	S	S	R	R	S	I	I

*R, Resistant; S, Sensitive; and I, Intermediate.*

### Adhesion Ability Results

An essential criterion for the selection of a probiotic is the ability to adhere to mucosal surfaces and epithelial cells, to allow its survival and colonization of the human gut. Hence the adhesion ability of probiotic candidates was examined using the Caco-2 cell line. Isolates SA 135, SA 171, SA 139, SA 12, ST 126, ST 172, ST 179, and ST 67 were able to adhere firmly to Caco-2 cell line while ST 80, SA 151, ST 109, ST 110, ST 16, and ST 13 showed moderate adhesion ability (Table 5).

**TABLE 5:** The adhesion ability of the selected isolates.

Strains	Adhesion	Strains	Adhesion
SA 135	Strong	ST 67	Strong
SA 171	Strong	ST 80	Moderate
SA 139	Strong	SA 151	Moderate
SA 12	Strong	SA 109	Moderate
ST 126	Strong	SA 110	Moderate
ST 172	Strong	SA 16	Moderate
ST 179	Strong	ST 13	Moderate

### 16s rRNA Sequencing and Phylogenetic Tree Results

**FIGURE 2:**
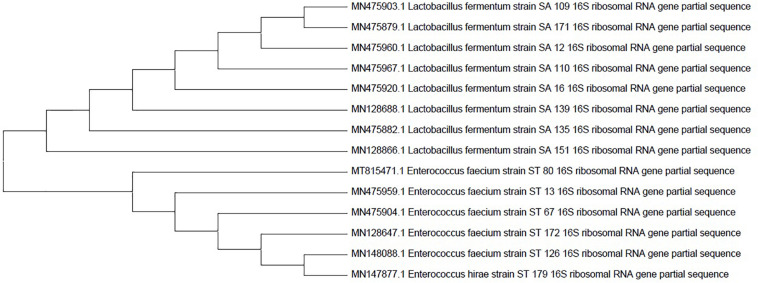
Phylogenetic tree of the 14 probiotic isolates based on 16s rRNA sequences. The tree was constructed by the MEGAX software using the bootstrap method.

Fourteen selected candidates were investigated for molecular characterization using the Sanger sequencing method and the BLAST tool. The Sanger sequencing data analysis and the resulting phylogenetic tree revealed that the isolates belong to *L. fermentum*, *Enterococcus faecium*, and *Enterococcus hirae* strains. All the isolates’ names and accession numbers can be found in Table 6. The phylogenetic tree was constructed by MEGAX software using the bootstrap method (Figure 2).

**TABLE 6:** Candidate probiotics identified based on percentage similarity of the 16s rRNA sequence to those available in the GenBank database.

Accession Number	Name	Similarity (%)	Strains
ST 80	84.44%	*Enterococcus faecium*	MT815471
SA 151	90.43%	*Lactobacillus fermentum*	MN128866
SA 135	98.30%	*Lactobacillus fermentum*	MN475882
SA 12	98.65%	*Lactobacillus fermentum*	MN475960
SA 139	98.93%	*Lactobacillus fermentum*	MN128688
ST 13	98.97%	*Enterococcus faecium*	MN475959
SA 109	99.00%	*Lactobacillus fermentum*	MN475903
ST 172	99.04%	*Enterococcus faecium*	MN128647
SA 171	99.15%	*Lactobacillus fermentum*	MN475879
SA 110	99.20%	*Lactobacillus fermentum*	MN475967
ST 179	99.35%	*Enterococcus hirae*	MN147877
SA 16	99.43%	*Lactobacillus fermentum*	MN475920
ST 126	99.71%	*Enterococcus faecium*	MN148088
ST 67	99.78%	*Enterococcus faecium*	MN475904

### Biofilm Production Results

All isolates showed strong biofilm formation except isolates SA 135, ST 13, ST 67, and ST 126, which only showed modest biofilm production.

### Auto-Aggregation and Hydrophobicity Results

Isolates were examined for adherence to hydrophobic surfaces, e.g., using xylene and the auto-aggregation method. As shown in Table 7, the isolates hydrophobicity extended from 0% to 69.68 ± 0.01% while auto-aggregation ranged from 2.23 ± 0.002% to 33.43 ± 0.007%. Maximum percentage of hydrophobicity to xylene was demonstrated by SA 135, followed by SA 151. In contrast, the highest auto-aggregation rate was observed in ST 179 and ST 13, respectively.

**TABLE 7:** Percentage of cell surface hydrophobicity of candidate probiotic strains.

Strains	Auto-aggregation (%)	Hydrophobicity (%)	Strains	Auto-aggregation (%)	Hydrophobicity (%)
SA 151	16.23 ± 0.002	55.84 ± 0.08	SA 16	15.77 ± 0.004	27.12 ± 0.007
ST 80	12.89 ± 0.002	20.69 ± 0.003	SA 110	6.29 ± 0.002	11.51 ± 0.04
SA 135	13.13 ± 0.004	69.68 ± 0.01	ST 13	17.39 ± 0.002	1.45 ± 0.01
SA 12	7.62 ± 0.006	41.14 ± 0.003	ST 172	11.30 ± 0.002	0
SA 171	2.23 ± 0.002	11.48 ± 0.007	ST 179	33.43 ± 0.007	6.44 ± 0.01
SA 139	11.81 ± 0.002	0	ST 67	13.82 ± 0.007	5.26 ± 0.01
SA 109	14.79 ± 0.005	32.24 ± 0.002	ST 126	10.57 ± 0.007	3 ± 0.01

*Values are shown in mean ± Standard deviation.*

### MTT Assay Results

**FIGURE 3:**
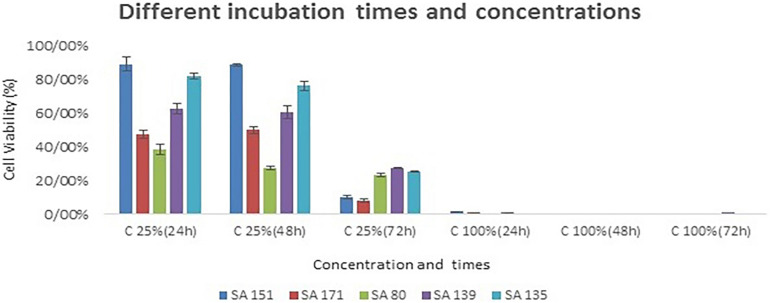
The cytotoxic effects of bacterial culture free supernatants on the CaCo-2 cell line at different supernatant concentrations and incubation periods. The five probiotic candidates that were assessed included SA 151, SA 171, SA139 and SA 135.

The top five isolates that exhibited relatively good probiotic properties were utilized for the MTT assay on Caco-2 cell line. As shown in Figure 3, the cytotoxic activities varied from 38% to 89% at 25% (v/v) concentration after 24 h of incubation, while boosting the concentration of supernatants to 100%, nearly all the cells were killed during the incubation period. By increasing the incubation period and the concentration of the supernatants, cytotoxic activity had fallen to 7% in the SA 171 isolate after 72 h of incubation. In general, isolates SA 171, ST 80 revealed the best cytotoxicity after 24 h of incubation.

## Discussion

Over the past decades, research in probiotics has gained a surge of interest because of their multiple health benefits and market demands. Many investigations have been undertaken to isolate new promising probiotic species from the human gut and salivary microbiota (Kiliç and Karahan, 2010; Vijayabharathi et al., 2012; Terai et al., 2015), but continuous research is required due to their species-specific features.

Probiotics used by humans are usually isolated from different environments that include dairy and non-dairy sources. However, probiotics that are isolated from human or animal intestines have certain characteristics that differ from those isolated from dairy products. For example, probiotics isolated from the human gut are usually more resistant to high bile salt concentrations and low pH levels. Furthermore, they possess higher adhering abilities when compared to those of dairy-isolated probiotics. Thus, non-diary probiotics are highly likely to be exploited in people who suffer from lactose intolerance (Sornplang and Piyadeatsoontorn, 2016; Sardana et al., 2018).

Traditional probiotics that have long been used globally, only cover a small spectrum of microorganisms. With the advent of next-generation sequencing, a greater understanding of the gut microbiome is revealing an immense number of new microorganisms with unknown characteristics that could have potential healing properties. The extensive research that is currently demonstrating the multidimensional benefits of the gut microbiome on human health will inevitably culminate in the identification and development of new microbial strains with novel therapeutic properties valuable to human health and the pharmaceutical industry. Accordingly, these new potential probiotic strains are referred to as “next-generation probiotics” (O’Toole et al., 2017).

This study seeks new probiotic strains, hence, lactic acid bacteria were isolated from the gut and saliva of healthy human individuals. In total, 185 isolated were tested for probiotic properties.

Before approving any probiotic for its health benefits and use in the food industry, its safety must be evaluated by *in vitro* and *in vivo* studies. The two main experimental tests that are carried out in this regard are the hemolysis and antibiotic resistance tests (Oh and Jung, 2015). None of the selected strains in this study showed beta-hemolytic activity.

The ability to tolerate harsh conditions, e.g., low pH, gastric juice, and bile salts, are the main contributing factors in the selection of good probiotic candidates (Kandylis et al., 2016). One of the key features in the selection of probiotics is acid tolerance, since they must be able to survive under the low pH conditions of the gastric juice in the stomach. In this study, the survival rate in acidic and bile salt circumstances vastly varied from one strain to another, suggesting a strain-specific pattern. Isolates SA 135, SA 151, and ST 80 showed maximum survival ability in the presence of acidic and bile salt conditions. The results of this study are in agreement with those of previous research (Chou and Weimer, 1999; Zago et al., 2011).

In our study, the inhibitory effects of LAB supernatants were found against a variety of pathogens including *S. aureus*, *P. aeruginosa*, MRSA, *E. coli*, *S. mutans*, *L. monocytogenes*, *B. cereus*, *E. faecalis*, and *C. albicans*. Our results differ somewhat from the published studies, whereby the LAB could not affect Gram-negative pathogens (Zommiti et al., 2018). These findings emphasize the importance of the selected strains in our studies, as they tend to show broad-spectrum antimicrobial activities, particularly against antibiotic-resistant microorganisms such as MRSA and the fungus, *C. albicans.* Although the broad antimicrobial effects of LABs are most often the result of organic acid production, the activity of antimicrobial peptides and other metabolites that may be produced by such strains cannot be ruled out (Kivanç et al., 2011; Somashekaraiah et al., 2019).

Another significant feature of probiotics is their ability to colonize the gut, which can be evaluated by the Caco-2 cell adhesion assay (Kozak et al., 2016), auto-aggregation, and hydrophobicity tests (Collado et al., 2008). In this research, the probiotic candidates showed a diverse adhesion model, demonstrating moderate to strong adhesion patterns. Regarding hydrophobicity and auto-aggregation, the highest rate of hydrophobicity and auto-aggregation were observed in SA 151, SA 135, and in ST 179, ST 13, respectively. The adhesion results demonstrate the ability of the selected strains to adhere to the epithelial cells and thus colonize the gut. Adhesion is a very important trait of a suitable and prevailing probiotic, as it prevents the colonization of the gastrointestinal tract by pathogenic bacteria (Abushelaibi et al., 2017; Somashekaraiah et al., 2019).

As a key feature, a good probiotic candidate should not possess or acquire any antibiotic resistance genes. Consistent with the literature, this research found that nearly all isolates were resistant to kanamycin and streptomycin, except for SA 109, which showed sensitivity to streptomycin (Kook et al., 2019). A possible explanation for these results may be the overuse of antibiotics in Iran. Notably, some were also found to be resistant to vancomycin, which is in accordance with previous reports that have shown vancomycin resistance as an intrinsic trait of LAB, such as *Lactobacillus, Leuconostoc*, and *Pediococcus*. In fact, many *Lactobacillus* strains, including *L. fermentum* are routinely used in the food industry. Vancomycin resistance in this group of bacteria is chromosomally encoded and is non-transferable and non-inducible (Swenson et al., 1990; Tynkkynen et al., 1998; Sharma et al., 2014).

Another important characteristic of the potential probiotic candidates in this study is their anti-cancer properties. Five of the selected isolates demonstrated antiproliferative activities against the human colon cancer cell line (Caco-2). Their culture supernatants inhibited the growth of cancerous cells by up to 7% after 72 h of incubation at the concentration of 25%. Our study confirms a previous finding by Lee et al. (2019), who demonstrated the antiproliferative effects of the culture-free supernatant (CFS) of a *L. fermentum* strain against colorectal cancer (CRC). It was shown that the CFS induces apoptosis, thereby inhibiting cell growth in CRC lines. The antiproliferative activity of *L. fermentum* is brought about by preventing NF-κB signaling. They showed that the *Lactobacillus* CFS is capable of inducing cell death, and thus has the potential to be used as a powerful multitarget anti-cancer chemotherapeutic agent (Lee et al., 2019).

In a previous study by Wei et al. (2019), it was suggested that exopolysaccharide (EPS) extracted from *L. fermentum* YL-11 could inhibit Caco-2 and HT-29 cell lines by up to 45.6 ± 6.1%. They proved that EPS could act as an antiproliferative agent in these two types of CRC cell lines (Wei et al., 2019).

Overall, we were able to select 14 potential probiotics with multifaceted probiotic attributes. These strains were subsequently characterized and identified genotypically, and were found to belong to the *Lactobacillus* and *Enterococcus* genus. By considering the unique characteristics of these indigenous probiotic strains, they can be of great benefit to the pharmaceutical, cosmetic, and food industries. Furthermore, this study also proved that the human gut and saliva can act as suitable sources of novel probiotics with desirable functional properties.

## Conclusion

Given the favorable probiotic attributes in our isolates, the following conclusion can be drawn that saliva and feces are two suitable and potential sources for isolating novel probiotics strains of human origin. Since the isolates of this study, in particular SA 151, ST 80, and SA 135, showed relatively good antipathogenic activity, survival in harsh conditions, biofilm production, and reasonable adhesion, they could, therefore, be viewed as promising “next generation” probiotic candidates, useful to the pharmaceutical industry.

## Data Availability Statement

The datasets presented in this study can be found in online repositories. The names of the repository/repositories and accession number(s) can be found in the article/supplementary material.

## Ethics Statement

The study involving human participants was reviewed and approved by the Ethics Committee at the National Institute of Genetic engineering and Biotechnology (NIGEB, Tehran, Iran), and was registered as IR.NIGEB.EC.1398.12.3.B. The patients/participants provided their written informed consent to participate in this study.

## Author Contributions

PS contributed to the original idea, conceptualization, supervision and project administration. HB and PS contributed to the design, methodology and implementation of research. PS and HB contributed to the writing and original draft preparation of the manuscript. PS contributed to the full revision and editing of the manuscript. HB, PS, SAJ, AA, and MB contributed to the analysis of the results and to the writing and editing of the manuscript.

## Conflict of Interest

The authors declare that the research was conducted in the absence of any commercial or financial relationships that could be construed as a potential conflict of interest.

## Footnotes

1 http://www.ncbi.nlm.nih.gov/GeneBank

## References

[B1] Abushelaibi A. Al-Mahadin S. El-Tarabily K. Shah N. P. Ayyash M. (2017). Characterization of potential probiotic lactic acid bacteria isolated from camel milk. *LWT Food Sci. Technol.* 79 316–325. 10.1016/j.lwt.2017.01.041

[B2] Alkalbani N. S. Turner M. S. Ayyash M. M. (2019). Isolation, identification, and potential probiotic characterization of isolated lactic acid bacteria and *in vitro* investigation of the cytotoxicity, antioxidant, and antidiabetic activities in fermented sausage. *Microb. Cell Fact* 18:188. 10.1186/s12934-019-1239-1 31690323 PMC6833168

[B3] Anwar F. Altayb H. N. Al-Abbasi F. A. Al-Malki A. L. Kamal M. A. Kumar V. (2020). Antiviral effects of probiotic metabolites on COVID-19. *J. Biomol. Struct. Dyn.* 1–10. 10.1080/07391102.2020.1775123 32475223 PMC7298884

[B4] Besser M. Terberger J. Weber L. Ghebremedhin B. Naumova E. A. Arnold W. H. (2019). Impact of probiotics on pathogen survival in an innovative human plasma biofilm model (hpBIOM). *J. Transl. Med.* 17:243. 10.1186/s12967-019-1990-4 31345229 PMC6659307

[B5] Borges S. Silva J. Teixeira P. (2012). Survival and biofilm formation by Group B *Streptococci* in simulated vaginal fluid at different pHs. *Antonie Van Leeuwenhoek Int. J. Gen. Mol. Microbiol.* 101 677–682. 10.1007/s10482-011-9666-y 22038130

[B6] Chen C. C. Lai C. C. Huang H. L. Huang W. Y. Toh H. S. Weng T. C. (2019). Antimicrobial activity of *Lactobacillus* species against carbapenem-resistant *Enterobacteriaceae*. *Front. Microbiol.* 10:789. 10.3389/fmicb.2019.00789 31057508 PMC6482263

[B7] Chen Z. Y. Hsieh Y. M. Huang C. C. Tsai C. C. (2017). Inhibitory effects of probiotic *Lactobacillus* on the growth of human colonic carcinoma cell line HT-29. *Molecules* 22:107. 10.3390/molecules22010107 28075415 PMC6155858

[B8] Chou L. S. Weimer B. (1999). Isolation and characterization of acid- and bile-tolerant isolates from strains of *Lactobacillus acidophilus*. *J. Dairy Sci.* 82 23–31. 10.3168/jds.S0022-0302(99)75204-5 10022003

[B9] Collado M. C. Meriluoto J. Salminen S. (2008). Adhesion and aggregation properties of probiotic and pathogen strains. *Eur. Food Res. Technol.* 226 1065–1073. 10.1007/s00217-007-0632-x

[B10] Daliri E. B. M. Lee B. H. Oh D. H. (2017). Current perspectives on antihypertensive probiotics. *Probiot. Antimicrob. Proteins* 9 91–101. 10.1007/s12602-016-9241-y 27900619

[B11] Dargahi N. Johnson G. Apostolopoulos V. (2020). *Streptococcus thermophilus* alters the expression of genes associated with innate and adaptive immunity in human peripheral blood mononuclear cells. *PLoS One* 15:e0228531. 10.1371/journal.pone.0228531 32045425 PMC7012395

[B12] d’Ettorre G. Ceccarelli G. Marazzato M. Campagna G. Pinacchio C. Alessandri F. (2020). Challenges in the management of SARS-CoV2 infection: the role of oral bacteriotherapy as complementary therapeutic strategy to avoid the progression of COVID-19. *Front. Med.* 7:389. 10.3389/fmed.2020.00389 32733907 PMC7358304

[B13] Drago L. (2019). Probiotics and colon cancer. *Microorganisms* 7:66. 10.3390/microorganisms7030066 30823471 PMC6463067

[B14] Ettinger G. MacDonald K. Reid G. Burton J. P. (2014). The influence of the human microbiome and probiotics on cardiovascular health. *Gut Microb.* 5 719–728. 10.4161/19490976.2014.983775 25529048 PMC4615746

[B15] FAO/WHO (2002). *Guidelines for the Evaluation of Probiotics in Food.* Rome: FAO.

[B16] Fernández M. F. Boris S. Barbés C. (2003). Probiotic properties of human *Lactobacilli* strains to be used in the gastrointestinal tract. *J. Appl. Microbiol.* 94 449–455. 10.1046/j.1365-2672.2003.01850.x 12588553

[B17] Fijan S. (2016). “Antimicrobial effect of probiotics against common pathogens,” in *Probiotics and Prebiotics in Human Nutrition and Health*, eds Rao V. Rao L. G. (London: InTechopen), 10.5772/63141

[B18] Ganapathy V. Thangaraju M. Prasad P. D. Martin P. M. Singh N. (2013). Transporters and receptors for short-chain fatty acids as the molecular link between colonic bacteria and the host. *Curr. Opin. Pharmacol.* 13 869–874. 10.1016/j.coph.2013.08.006 23978504

[B19] Gao C. Ganesh B. P. Shi Z. Shah R. R. Fultz R. Major A. (2017). Gut microbe-mediated suppression of inflammation-associated colon carcinogenesis by luminal histamine production. *Am. J. Pathol.* 187 2323–2336. 10.1016/j.ajpath.2017.06.011 28917668 PMC5809336

[B20] Gill H. S. Rutherfurd K. J. Prasad J. Gopal P. K. (2000). Enhancement of natural and acquired immunity by *Lactobacillus rhamnosus* (HN001), *Lactobacillus acidophilus* (HN017) and *Bifidobacterium lactis* (HN019). *Br. J. Nutr.* 83 167–176. 10.1017/s0007114500000210 10743496

[B21] Gómez-Guzmán M. Toral M. Romero M. Jiménez R. Galindo P. Sánchez M. (2015). Antihypertensive effects of probiotics *Lactobacillus* strains in spontaneously hypertensive rats. *Mol. Nutr. Food Res.* 59 2326–2336. 10.1002/mnfr.201500290 26255877

[B22] Haghshenas B. Haghshenas M. Nami Y. Khosroushahi A. Y. Abdullah N. Barzegari A. (2016). Probiotic assessment of *Lactobacillus plantarum* 15HN and *Enterococcus mundtii* 50H isolated from traditional dairies microbiota. *Adv. Pharm. Bull.* 6:37. 10.15171/APB.2016.007 27123416 PMC4845554

[B23] Haghshenas B. Nami Y. Abdullah N. Radiah D. Rosli R. Khosroushahi A. Y. (2014). Anti-proliferative effects of *Enterococcus* strains isolated from fermented dairy products on different cancer cell lines. *J. Funct. Foods* 11 363–374. 10.1016/j.jff.2014.10.002

[B24] Halder D. Mandal M. Chatterjee S. S. Pal N. K. Mandal S. (2017). Indigenous probiotic *Lactobacillus* isolates presenting antibiotic like activity against human pathogenic bacteria. *Biomedicines* 5:31. 10.3390/biomedicines5020031 28621711 PMC5489817

[B25] Kahouli I. Malhotra M. Westfall S. Alaoui-Jamali M. A. Prakash S. (2017). Design and validation of an orally administrated active *L. fermentum*-*L. acidophilus* probiotic formulation using colorectal cancer Apc ^*Min/+*^ mouse model. *Appl. Microbiol. Biotechnol.* 101 1999–2019. 10.1007/s00253-016-7885-x 27837314

[B26] Kandylis P. Pissaridi K. Bekatorou A. Kanellaki M. Koutinas A. A. (2016). Dairy and non-dairy probiotic beverages. *Curr. Opin. Food Sci.* 7 58–63. 10.1016/j.cofs.2015.11.012

[B27] Kang M.-S. Lim H.-S. Oh J.-S. Lim Y.-J. Wuertz-Kozak K. Harro J. M. (2017). Antimicrobial activity of *Lactobacillus salivarius* and *Lactobacillus fermentum* against *Staphylococcus aureus*. *Pathog. Dis.* 75:ftx009. 10.1093/femspd/ftx009 28158586

[B28] Kiliç G. B. Karahan A. G. (2010). Identification of lactic acid bacteria isolated from the fecal samples of healthy humans and patients with dyspepsia, and determination of their pH, bile, and antibiotic tolerance properties. *J. Mol. Microbiol. Biotechnol.* 18 220–229. 10.1159/000319597 20668388

[B29] Kivanç M. Yilmaz M. Çakir E. (2011). Isolation and identification of lactic acid bacteria from boza, and their microbial activity against several reporter strains. *Turkish J. Biol.* 35 313–324. 10.3906/biy-0906-67 31411186

[B30] Kocsis T. Molnár B. Németh D. Hegyi P. Szakács Z. Bálint A. (2020). Probiotics have beneficial metabolic effects in patients with type 2 diabetes mellitus: a meta-analysis of randomized clinical trials. *Sci. Rep.* 10:11787. 10.1038/s41598-020-68440-1 32678128 PMC7366719

[B31] Kook S.-Y. Chung E.-C. Lee Y. Lee D. W. Kim S. (2019). Isolation and characterization of five novel probiotic strains from Korean infant and children faeces. *PLoS One* 14:e0223913. 10.1371/journal.pone.0223913 31671118 PMC6822945

[B32] Kozak K. Charbonneau D. Sanozky-Dawes R. Klaenhammer T. (2016). Characterization of bacterial isolates from the microbiota of mothers’ breast milk and their infants. *Gut Microb.* 6 341–351. 10.1080/19490976.2015.1103425 26727418 PMC4826109

[B33] Krausova G. Hyrslova I. Hynstova I. (2019). *In Vitro* evaluation of adhesion capacity, hydrophobicity, and auto-aggregation of newly isolated potential probiotic strains. *Fermentation* 5:100 10.3390/fermentation5040100

[B34] Kumar M. Dhaka P. Vijay D. Vergis J. Mohan V. Kumar A. (2016). Antimicrobial effects of *Lactobacillus plantarum* and *Lactobacillus acidophilus* against multidrug-resistant enteroaggregative *Escherichia coli*. *Int. J. Antimicrob. Agents* 48 265–270. 10.1016/j.ijantimicag.2016.05.014 27451088

[B35] Kumar R. Sood U. Gupta V. Singh M. Scaria J. Lal R. (2020). Recent advancements in the development of modern probiotics for restoring human gut microbiome *Dysbiosis*. *Indian J. Microbiol.* 60 12–25. 10.1007/s12088-019-00808-y 32089570 PMC7000592

[B36] Lee J.-E. Lee J. Kim J. H. Cho N. Lee S. H. Park S. B. (2019). Characterization of the anti-cancer activity of the probiotic bacterium *Lactobacillus fermentum* using 2D vs. 3D culture in colorectal cancer cells. *Biomolecules* 9:557. 10.3390/biom9100557 31581581 PMC6843223

[B37] McFarland L. (2015). Probiotics for the primary and secondary prevention of *C. difficile* infections: a meta-analysis and systematic review. *Antibiotics* 4 160–178. 10.3390/antibiotics4020160 27025619 PMC4790329

[B38] Nami Y. Haghshenas B. Haghshenas M. Abdullah N. Khosroushahi A. Y. (2015). The Prophylactic effect of probiotic *Enterococcus lactis* IW5 against different human cancer cells. *Front. Microbiol.* 6:1317. 10.3389/fmicb.2015.01317 26635778 PMC4659899

[B39] Nami Y. Vaseghi Bakhshayesh R. Manafi M. Hejazi M. A. (2019a). Hypocholesterolaemic activity of a novel autochthonous potential probiotic *Lactobacillus plantarum* YS5 isolated from yogurt. *LWT Food Sci. Technol.* 111 876–882. 10.1016/j.lwt.2019.05.057

[B40] Nami Y. Vaseghi Bakhshayesh R. Mohammadzadeh Jalaly H. Lotfi H. Eslami S. Hejazi M. A. (2019b). Probiotic properties of *Enterococcus* isolated from artisanal dairy products. *Front. Microbiol.* 10:300. 10.3389/fmicb.2019.00300 30863379 PMC6400110

[B41] Oh Y. J. Jung D. S. (2015). Evaluation of probiotic properties of *Lactobacillus* and *Pediococcus* strains isolated from omegisool, a traditionally fermented milletalcoholic beverage in Korea. *LWT Food Sci. Technol.* 63 437–444. 10.1016/j.lwt.2015.03.005

[B42] O’Toole P. W. Marchesi J. R. Hill C. (2017). Next-generation probiotics: the spectrum from probiotics to live biotherapeutics. *Nat. Microbiol.* 2:17057. 10.1038/nmicrobiol.2017.57 28440276

[B43] Pérez Ibarreche M. Castellano P. Vignolo G. (2014). Evaluation of anti-Listeria meat borne *Lactobacillus* for biofilm formation on selected abiotic surfaces. *Meat Sci.* 96 295–303. 10.1016/j.meatsci.2013.07.010 23933630

[B44] Plaza-Díaz J. Ruiz-Ojeda F. J. Vilchez-Padial L. M. Gil A. (2017). Evidence of the anti-inflammatory effects of probiotics and synbiotics in intestinal chronic diseases. *Nutrients* 9:555. 10.3390/nu9060555 28555037 PMC5490534

[B45] Sanders M. E. (2008). Probiotics: definition, sources, selection, and uses. *Clin. Infect. Dis.* 46 S58–S61. 10.1086/523341 18181724

[B46] Sardana R. K. Chhikara N. Tanwa B. Pangh A. (2018). Dietary impact on esophageal cancer in humans: a review. *Food Funct.* 9 1967–1977. 10.1039/c7fo01908d 29616242

[B47] Sharma P. Tomar S. K. Goswami P. Sangwan V. Singh R. (2014). Antibiotic resistance among commercially available probiotics. *Food Res. Int.* 57 176–195. 10.1016/j.foodres.2014.01.025

[B48] Silva D. R. Sardi J. C. O. de Pitangui N. S. Roque S. M. da Silva A. C. B. Rosalen P. L. (2020). Probiotics as an alternative antimicrobial therapy: current reality and future directions. *J. Funct. Foods* 73:104080 10.1016/j.jff.2020.104080

[B49] Singh N. Gurav A. Sivaprakasam S. Brady E. Padia R. Shi H. (2014). Activation of Gpr109a, receptor for niacin and the commensal metabolite butyrate, suppresses colonic inflammation and carcinogenesis. *Immunity* 40 128–139. 10.1016/j.immuni.2013.12.007 24412617 PMC4305274

[B50] Somashekaraiah R. Shruthi B. Deepthi B. V. Sreenivasa M. Y. (2019). Probiotic properties of lactic acid bacteria isolated from neera: a naturally fermenting coconut palm nectar. *Front. Microbiol.* 10:1382. 10.3389/fmicb.2019.01382 31316477 PMC6611078

[B51] Sornplang P. Piyadeatsoontorn S. (2016). Probiotic isolates from unconventional sources: a review. *J. Anim. Sci. Technol.* 58:26. 10.1186/s40781-016-0108-2 27437119 PMC4949924

[B52] Swenson J. M. Facklam R. R. Thornsberry C. (1990). Antimicrobial susceptibility of vancomycin-resistant *Leuconostoc*, *Pediococcus*, and *Lactobacillus* species. *Antimicrob. Agents Chemother.* 34 543–549. 10.1128/AAC.34.4.543 2344161 PMC171641

[B53] Tan G. S. E. Tay H. L. Tan S. H. Lee T. H. Ng T. M. Lye D. C. (2020). Gut microbiota modulation: implications for infection control and antimicrobial stewardship. *Adv. Ther.* 37 4054–4067. 10.1007/s12325-020-01458-z 32767183 PMC7412295

[B54] Terai T. Okumura T. Imai S. Nakao M. Yamaji K. Ito M. (2015). Screening of probiotic candidates in human oral bacteria for the prevention of dental disease. *PLoS One* 10:e0128657. 10.1371/journal.pone.0128657 26053410 PMC4459870

[B55] Tynkkynen S. Singh K. V. Varmanen P. (1998). Vancomycin resistance factor of *Lactobacillus rhamnosus* GG in relation to enterococcal vancomycin resistance (van) genes. *Int. J. Food Microbiol.* 41 195–204. 10.1016/S0168-1605(98)00051-8 9706787

[B56] Valdéz J. C. Peral M. C. Rachid M. Santana M. Perdigón G. (2005). Interference of *Lactobacillus plantarum* with *Pseudomonas aeruginosa* in vitro and in infected burns: the potential use of probiotics in wound treatment. *Clin. Microbiol. Infect.* 11 472–479. 10.1111/j.1469-0691.2005.01142.x 15882197

[B57] Vernazza C. L. Gibson G. R. Rastall R. A. (2006). Carbohydrate preference, acid tolerance and bile tolerance in five strains of *Bifidobacterium*. *J. Appl. Microbiol.* 100 846–853. 10.1111/j.1365-2672.2006.02832.x 16553741

[B58] Vijayabharathi R. Palanisamy B. D. Manoharan R. K. Sathyabama S. Bruntha P. Priyadarisini V. B. (2012). Screening for probiotic properties of strains isolated from feces of various human groups. *Artic. J. Microbiol.* 50 603–612. 10.1007/s12275-012-2045-1 22923108

[B59] Vinderola C. G. Medici M. Perdigón G. (2004). Relationship between interaction sites in the gut, hydrophobicity, mucosal immunomodulating capacities and cell wall protein profiles in indigenous and exogenous bacteria. *J. Appl. Microbiol.* 96 230–243. 10.1046/j.1365-2672.2004.02158.x 14723684

[B60] Wei Y. Li F. Li L. Huang L. Li Q. (2019). Genetic and biochemical characterization of an exopolysaccharide with in vitro antitumoral activity produced by *Lactobacillus fermentum* YL-11. *Front. Microbiol.* 10:2898. 10.3389/fmicb.2019.02898 31921073 PMC6929415

[B61] Westfall S. Lomis N. Kahouli I. Dia S. Y. Singh S. P. Prakash S. (2017). Microbiome, probiotics and neurodegenerative diseases: deciphering the gut brain axis. *Cell. Mol. Life Sci.* 74 3769–3787. 10.1007/s00018-017-2550-9 28643167 PMC11107790

[B62] Xu H. Jeong H. S. Lee H. Y. Ahn J. (2009). Assessment of cell surface properties and adhesion potential of selected probiotic strains. *Lett. Appl. Microbiol.* 49 434–442. 10.1111/j.1472-765X.2009.02684.x 19725886

[B63] Zago M. Fornasari M. E. Carminati D. Burns P. Suàrez V. Vinderola G. (2011). Characterization and probiotic potential of *Lactobacillus plantarum* strains isolated from cheeses. *Food Microbiol.* 28 1033–1040. 10.1016/j.fm.2011.02.009 21569949

[B64] Zommiti M. Cambronel M. Maillot O. Barreau M. Sebei K. Feuilloley M. (2018). Evaluation of probiotic properties and safety of *Enterococcus faecium* isolated from artisanal Tunisian Meat “Dried Ossban.”. *Front. Microbiol.* 9:1685. 10.3389/fmicb.2018.01685 30127770 PMC6088202

